# MetaNetVar: Pipeline for applying network analysis tools for genomic variants analysis

**DOI:** 10.12688/f1000research.8288.1

**Published:** 2016-04-13

**Authors:** Eric Moyer, Megan Hagenauer, Matthew Lesko, Felix Francis, Oscar Rodriguez, Vijayaraj Nagarajan, Vojtech Huser, Ben Busby

**Affiliations:** 1 National Center for Biotechnology Information, Bethesda, USA; 2 Molecular, Behavioral Neuroscience Institute, University of Michigan, Ann Arbor, USA; 3 National Center for Biotechnology Information, National Library of Medicine, Bethesda, USA; 4 Bioinformatics and Systems Biology program, University of Delaware, Newark, USA; 5 Department of Genetics and Genomic Sciences, Icahn School of Medicine at Mount Sinai, New York, USA; 6 Bioinformatics and Computational Biosciences Branch, National Institute of Allergy and Infectious Diseases, National Institute of Mental Health, Bethesda, USA; 7 Lister Hill National Center for Biomedical Communications, National Library of Medicine, National Institute of Mental Health, Bethesda, USA

**Keywords:** network analysis, genetic variant, pipeline, next generation sequencing

## Abstract

Network analysis can make variant analysis better. There are existing tools like HotNet2 and dmGWAS that can provide various analytical methods. We developed a prototype of a pipeline called MetaNetVar that allows execution of multiple tools. The code is published at https://github.com/NCBI-Hackathons/Network_SNPs. A working prototype is published as an Amazon Machine Image - ami-4510312f .

## Introduction

Traditionally, the goal of genome-wide association studies (GWAS) has been to associate single nucleotide polymorphisms (SNPs) and their respective haplotype blocks with disease status, allowing the eventual identification of particular genes responsible for disease phenotype. Unfortunately, only a small subset of diseases arise from variants within a single gene. For most complex diseases, it is likely that the disease arises due to the interactive effects of multiple genetic variants, and different collections of these variants may be present in different patients. Within a GWAS study, these variants individually will exhibit low predictive power making it difficult for researchers to obtain a sufficient sample size to identify them with high confidence. Therefore, tools that can help detect groups of interacting genetic variants are needed ^1– 3^.

One set of tools that has great potential for aiding in this problem is network analyses. Within these tools, the results from GWAS studies are overlaid on networks constructed from curated molecular interaction data, such as databases documenting protein-protein interactions (PPIs), protein-DNA interactions, metabolite interactions, and gene-gene co-expression ^1, 2^. Many of these tools are powerful, but somewhat inaccessible to users with weaker computational backgrounds. For example, installing, configuring, running, and comparing the output of multiple network analysis tools could require a working knowledge of command-line scripting, Python, R, and Perl. Therefore, the goal of our hackathon team was to create a single command-line pipeline within which a user could input the results of a GWAS study, execute existing network analysis tools, and then access results from multiple network analyses. This work was conducted as part of the NCBI January 2016 Hackathon.

## Methods

The context of the hackathon event allowed only three development days to create the pipeline which impacted the scope and design of the tool. The focus was on allowing one input file to be directed towards multiple tools; consolidation of results from individual tools was out of scope. Similarly, each tool output was not post-processed for unified output. We envision that future improvement to the pipeline may offer advanced visualisation options; however, this was not part of this pilot implementation. A working instance of the pipeline is also published as an Amazon Machine Image ami-4510312f.

### Tools used in the pipeline

As much as possible, the MetaNetVar pipeline uses existing tools for network analysis. We only considered tools that are freely available, with no license restrictions. We describe briefly each tool that is integrated into the MetaNetVar pipeline. Tools vary in scope, and some include additional functions that include network analysis.

### FunSeq2 (Version 2.1.2)

FunSeq2 is an existing tool for prioritizing variants using several different approaches, including network-based analysis ^4, 5^. FunSeq2 identifies hub genes and provides the measure of centrality for those hub genes. Inference of the network analysis results requires further processing of the program’s output. We chose to include FunSeq2 in our pipeline because of its capability to identify functionally important, non-coding variants in the context of biological networks.

### NetworkX (Version 1.10)

NetworkX is a network analysis framework available in a Python language software package. It allows for “the creation, manipulation, and study of the structure, dynamics, and functions of complex networks” ^6^. It contains many standard graph algorithms and accepts and outputs 13 file formats, where nodes can be anything and edges can hold any type of data. NetworkX was used to calculate the degrees and betweenness centrality of nodes (genes) and to create XML format and static PNG figures of subnetworks containing the input genes. The degrees and the betweenness centrality gives you a measurement of how important the gene is in the network. A network analysis framework similar to NetworkX is CytoscapeJS ^7^. We chose to include NetworkX in our pipeline because of our experience with Python.

### HotNet2 (Version 1.0.1)

HotNet2 is an algorithm for detecting “significantly altered subnetworks in a large gene interaction network” ^8– 10^. The algorithm uses heat diffusion kernel to capture the local topology of the interaction network. The subnetworks in genome scale interactions that have non-random mutations are identified using this approach. The limitation of HotNet2 are the challenges in getting the scripts running straight out of the box, along with the long computational time involved in the preliminary influence matrix creation process. We chose to include HotNet2 in our pipeline because of our experience with Python.

### dmGWAS (Version 3.0)

dmGWAS_3.0 is an existing tool for overlaying gene-level summaries of case-control association p-values onto an existing network (in this case, we use the network extracted from GeneMania detailed below) and then identifying subnetworks that are particularly enriched for strong associations using a greedy algorithm ^11, 12^. Unlike the previous version of dmGWAS (2.0), dmGWAS_3.0 also has the ability to incorporate differential gene coexpression data (in other words, the difference in gene co-expression between cases and controls) as weights for the edges in the network, but for the sake of simplicity we did not make use of this new functionality in our pipeline. Due to this choice, we discovered that the dense-module search output (ResList.RData) took the format of the previous version dmGWAS_2.0 ^13^ and could not be manipulated using the tools referenced in the current documentation. Therefore, we created our own short script to extract out the basic statistics and subnetwork nodes associated with each input gene present in the network (see below: ModuleStrengthSummaryByGene.txt and Top1000ModuleScores.txt). We later discovered that some of the old tools capable of manipulating dmGWAS_2.0 output (ResList.RData) were preserved in the current code package and could be used for further data exploration by a motivated user by loading the output file (ResList.RData) into R and installing the requisite packages (dmGWAS, igraph), although some of the tools did not appear to be fully functional anymore (such as the subnetwork plotting capability in simpleChoose()).

Overall, the primary limitation that we observed for dmGWAS was computing time, so we adapted the existing code to make use of parallel computing using the BiocParallel package in R ^14^.

To utilize dmGWAS_3.0, it is first necessary to convert the input file containing the case-control association p-values for each SNP to a gene-level summary. Within our pipeline, we complete this conversion using VEGAS, an existing command line (Linux/Unix) based tool recommended within the dmGWAS documentation ^15, 16^. It should be noted that by default, VEGAS uses the HapMap2 CEU (Central Europeans, Utah) population to estimate patterns of linkage disequilibrium for each gene.

VEGAS is written in Perl but also makes use of two R packages (corpcor and mvtnorm) and depends on functions provided by PLINK, a commonly-used whole genome analysis toolset ^17, 18^. The output of VEGAS requires further processing before input into dmGWAS. We found that several of the VEGAS gene-level p-values were rounded to either 0 or 1, which was incompatible with dmGWAS, so we substituted the minimum p-value present in our test file (1e-06) for 0 and 0.999 for 1.

Table 1 provides a summary of the tools used in this pipeline, including notes about their advantages and disadvantages.

**Table 1.** An overview of the tools used in our pipeline.

Name	Advantages	Disadvantages	Platform
FunSeq2	Uses ENCODE Regulatory Network data to identify hubs	Output needs to be parsed to better understand the network related results; make sure the correct reference build and the correct coordinate system (inclusive or exclusive) is used	Perl program
NetworkX	Ease-of-use, rapid development, open-source, flexible graph implementations	Cannot use for large-scale problems with more than 100 million nodes	Python library
HotNet2	HotNet2 algorithm uses heat diffusion kernel analogous to random walk with restart to better capture the local topology of the interaction network.	Challenging to run the scripts directly; poor documentation; had to fix some bugs to get it working; the one time influence matrix creation for a new network may take several hours.	Python
dmGWAS	Predicts molecular subnetworks that are enriched for disease- associations using the full results from a GWAS study (no thresholding of input by p-value or rank!).	Computationally intensive: may take days to produce results. Our updated version of dmGWAS uses parallel computing to speed up processing time, but still may take several hours even on a large cloud server. Some syntax provided in the documentation does not function, and output contents follow an older format. However, only minimal tweaking was required for dmGWAS to be integrated into the pipeline.	R package (but dependent on command- line VEGAS and PLINK toolsets)

## Networks used

Network construction based on a user-provided list of variants required accessing molecular interaction network data from external databases. We describe the network databases utilized by each tool. Some networks (such as Multinet) are used by multiple tools (FunSeq2 and HotNet2).

### FunSeq2

FunSeq2 utilizes multinet ^19^, which is an integrated network consisting of regulatory interactions from the ENCODE regulatory network ^20, 21^, phosphorylation interactions from the SignaLink database ^22– 24^, protein-protein interactions from BioGRID (release 3.1.83) ^25^, and metabolic interactions from KEGG ^26^. While there are options for users to bring in their own network for use with FunSeq2 analysis, this pipeline prototype uses the pre-packaged multinet.

### NetworkX & dmGWAS

The NetworkX and dmGWAS are libraries and do not include particular network data.

We paired GeneMania with NetworkX and dmGWAS. The GeneMania network is a protein-protein interaction network ^27, 28^. Two genes are connected if they are found to interact in a protein-protein interaction study. The network was created from various protein-protein interaction databases, including BioGRID and Pathway Commons ^29, 30^. We used version 2014-10-15 of Homo_sapiens.COMBINED network.

### HotNet2

HotNet2 uses mutation data to prioritize subnetworks by identifying significantly mutated subnetworks in genome scale interaction networks. In our pipeline, we have used the 2012 version of HINT (High-quality INTeractomes) a database of high-quality protein-protein interactions ^31^.

### Example data

As a sample input for our pilot, we searched NCBI dbGaP for a sample study that provided a real world list of variants. We used data from a clinical study of age-related macular degeneration ^32^ with dbGAP identifier phs000182.v3.p1.

As an additional input example, we used data from ClinVar ^33^. ClinVar is a database of interpretations of clinical significance of variants for reported conditions, hosted by the National Center for Biotechnology Information (NCBI). It includes germline and somatic variants of any size, type, or genomic location with interpretations from several sources (such as clinical testing laboratories, research laboratories, or locus-specific databases). It includes a link of variants to phenotypes. For this example, we identified variations submitted by LabCorp and extracted disease-variant pairs, for diseases with 30+ variants. The example dataset is provided on the MetaNetVar GitHub page.

## Results

**Figure 1.**
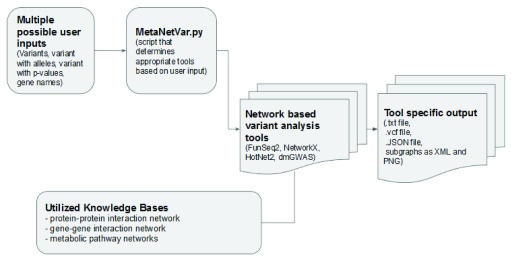
An overview of the pipeline.

We implemented four network analysis programs or platforms into our pipeline (FunSeq2, NetworkX, HotNet2, dmGWAS), utilizing molecular interaction data from several external knowledge databases (listed above). Figure 1 provides an overview of the pipeline.

To lower the adoption threshold for potential users, we offer the snapshot of our working instance as an Amazon Machine Image. The collection of tools and the pipeline script can be executed using an instance of our publicly available Amazon Machine Image: ami-4510312f. The accompanying supplementary file describes the step-by-step procedure for running our pipeline using the published Amazon Machine Image.

We discuss below the results from individual tools integrated into our pipeline. Results of all of these tools, using the example dataset, is also provided on the MetaNetVar GitHub page.

### FunSeq2

The parsed input file required for the FunSeq2 analysis, the PHP script that generates this parsed input file from the original dbGaP association data, and the output files (using default parameters) are provided in the GitHub project page. An example file generated from a filtered list of SNPs from the ClinVar database, for the Cardiomyopathy phenotype is also provided for testing purposes and can be found at http://github.com/NCBI-Hackathons/Network_SNPs/blob/master/test/sample_output/funseq2/cardiomyopathyfunseqoutput.

### NetworkX

**Figure 2.**
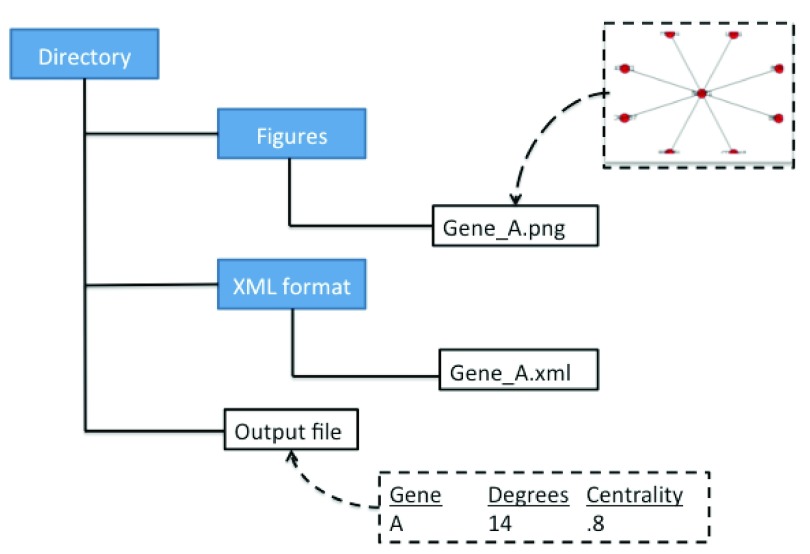
NetworkX outputs a file containing the degreeness and centrality of each gene, as well as two directories containing subnetwork graph figures for each input gene (.png) and its XML format.

We took the NetworkX library and created a script that we refer to as SNPsNet. This script generates one output file containing the degrees and betweenness centrality measure of genes that are input into the pipeline, as well as creating two directories (see Figure 2). The two directories contain figures of subnetworks with the input genes and the XML format of the subnetworks. With these results, the user can prioritize the input variants or genes by sorting how important each gene is based on degreeness or centrality, as well as visualizing the subnetwork. Since NetworkX is not primarily a visualization tool, the XML file can be input into several other tools to better visualize the graph.

### HotNet2

The influence matrix for HINT was pre-computed and then used in the current version of our pipeline. Influence matrix creation is a one-time process for a given network and, if required, advanced users may use custom influence matrices with MetaNetVar by modifying the path to the input influence matrix file and corresponding gene index file. For evaluation of MetaNetVar, we generated heat scores from a test mutation file. The .json file containing heat scores on each gene, which was used in subsequent steps, may be accessed at https://github.com/NCBI-Hackathons/Network_SNPs/blob/master/heat_score.json.

The final step of weighted graph generation uses the influence matrix for HINT, the HINT index file, and the heat score .json file, to remove edges with weight less than the delta value, and extract the resulting connected components. Two output files were generated: components.txt (available at https://github.com/NCBI-Hackathons/Network_SNPs/blob/master/components.txt) and results.json (available at https://github.com/NCBI-Hackathons/Network_SNPs/blob/master/results.json)

### dmGWAS

**Figure 3.**
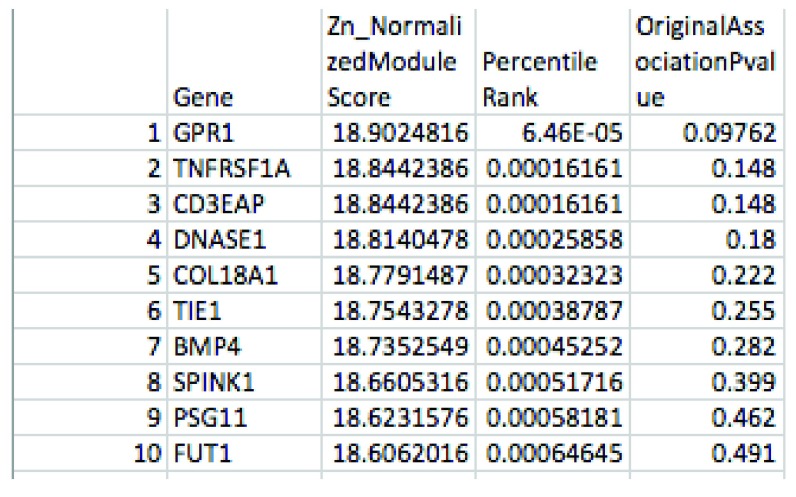
An example of the first output summary file produced by dmGWAS in our pipeline: ModuleStrengthSummaryByGene.txt. This file provides the Normalized Module Score for each gene included in the network (“Zn”, where a larger value indicates the gene is more enriched for significant case-control associations), and the gene-level summary case-control association p-value provided by VEGAS. It is ordered by percentile rank to allow comparison across different network analysis programs.

**Figure 4.**
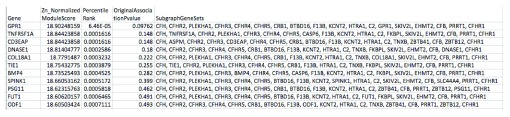
An example of the second output summary file produced by dmGWAS in our pipeline: Top1000ModuleScores.txt. This second output provides similar information as the first output file, but expands it to include the list of genes (nodes) present in each gene of interest subnetwork. Only subnetwork output for the top 1000 seed genes is provided (as determined by percentile rank).

The sample association file from the age-related macular degeneration dataset (phs000182) was parsed down to a two-column text file containing only SNP identifiers (“rs numbers”) and case-control association p-values. This file was fed into VEGAS and a gene-level summary file was created, which was further parsed into a simple two-column text file containing gene identifiers (gene symbol) and “weight” (an integrated p-value for the gene). Within dmGWAS, this input was overlaid onto a network provided by GeneMania to produce a network of weighted nodes from which particularly “dense” subnetworks are identified (full output: ResList.RData). Finally, our program summarizes the data into two easily navigable tab-delimited .txt files which can be viewed within accessible programs such as Microsoft Excel (ModuleStrengthSummaryByGene.txt, Top1000ModuleScores.txt). Figure 3 and Figure 4 demonstrate example output files.

### Limitations

The current version of the pipeline is set to use data from dbGaP and ClinVar out-of-the-box. However, advanced users could tweak the provided scripts to make it run using other input formats. Some of the components of the pipeline use processes that are parallel and compute-intensive in nature. Using the provided working implementation of the pipeline through Amazon Web Services requires some computing skills.

## Conclusions

Our tool, MetaNetVar, allows researchers with limited computational experience to access a host of powerful network analysis tools for application to genomic datasets. This platform is intended for use in a variety of future hackathons, including work on cancer and evolutionary biology, but will most likely also be used by participants from the current hackathon, as well as other interested individuals. Since this work was a pilot project, we expect further modification of the pipeline as new users provide feedback. Ideally, the future pipeline would include a unified output summary, better network visualization tools, and the ability to integrate known disease-related variants into the analysis, such as from ClinVar ^33^, from PheGeni ^34^, or from the output of epistasis analyses ^35^.

## Data and software availability

Latest source code: https://github.com/NCBI-Hackathons/Network_SNPs

Archived source code as at time of publication: http://dx.doi.org/10.5281/zenodo.48202 ^36^

Amazon instance ID: ami-4510312f

Amazon instance name: NCBI-Hackathon-20160122-Network-SNPs

License: CC0 1.0 Universal

## Supplementary material

Software manual. This document provides instructions on how to start and run the NCBI-Hackathon-20160122-Network-SNPs instance in AWS using a Mac computer.
