# Supplementary material for: MetaNetVar: Pipeline for applying network analysis tools for genomic variants analysis
Source: F1000Res. 2016 Apr 13;5:674. [Version 1] doi: 10.12688/f1000research.8288.1 (PMC4857755; doi:10.12688/f1000research.8288.1)

**Supplement One: Software Manual**

This document provides instructions on how to start and run the NCBI-Hackathon-20160122-Network-SNPs instance in AWS using a Mac computer.

1. Visit aws.amazon.com


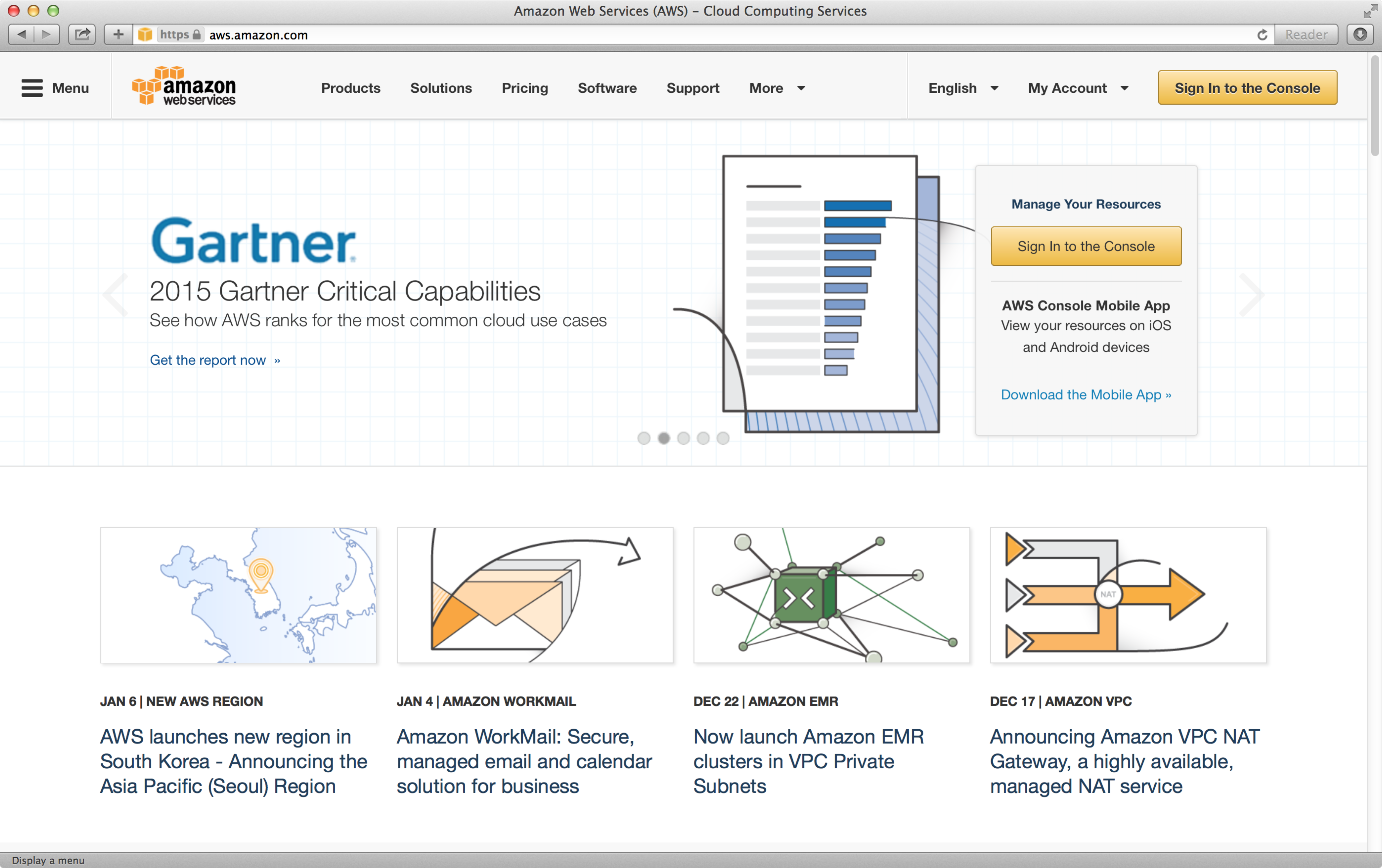


2. Click the “Sign in to the Console”
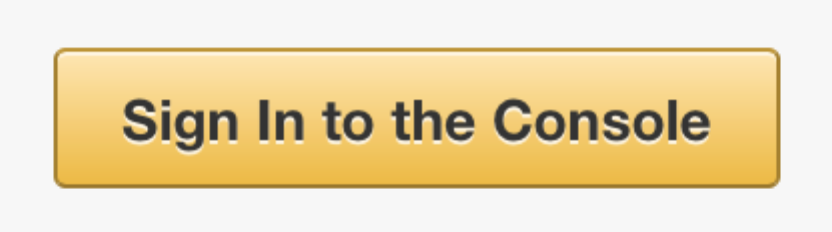
button to login. If you don’t have an account already, register for one and then login to your account.


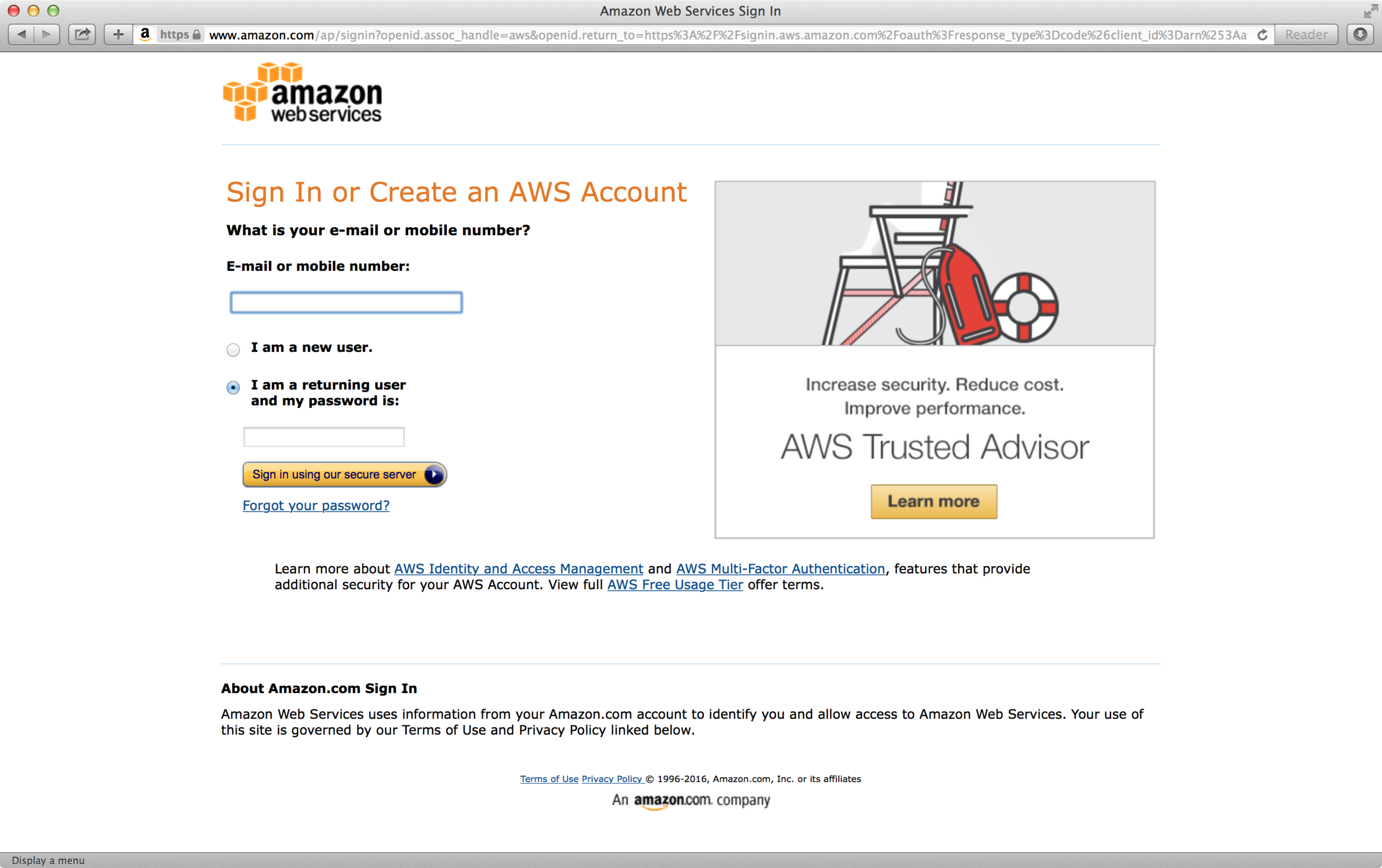


3. Once you are in, click the “EC2” icon
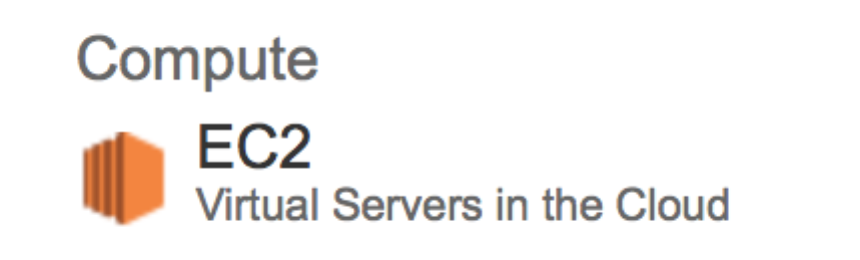

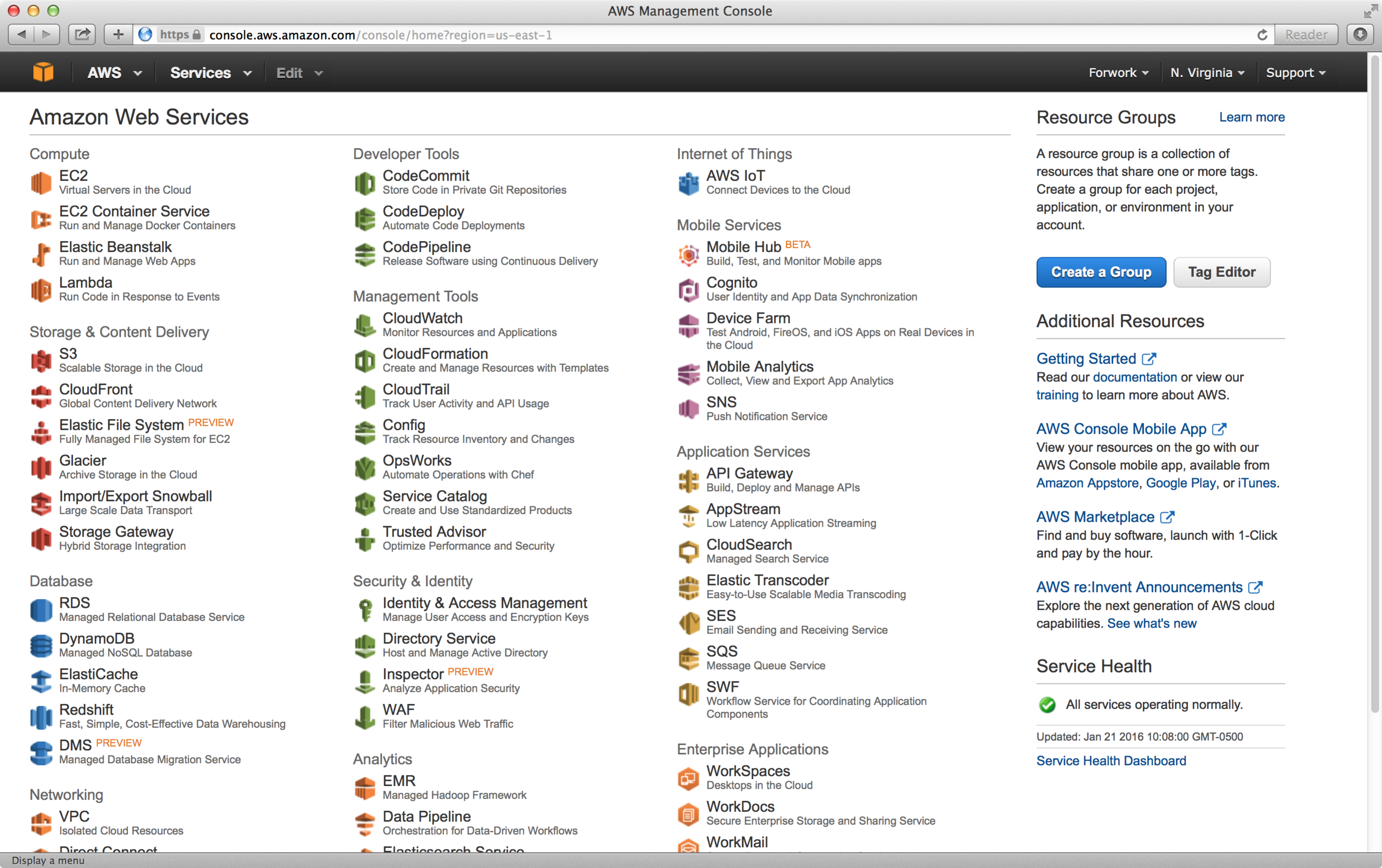


4. Once you are in the EC2 dashboard, choose “US East (N. Virginia)”
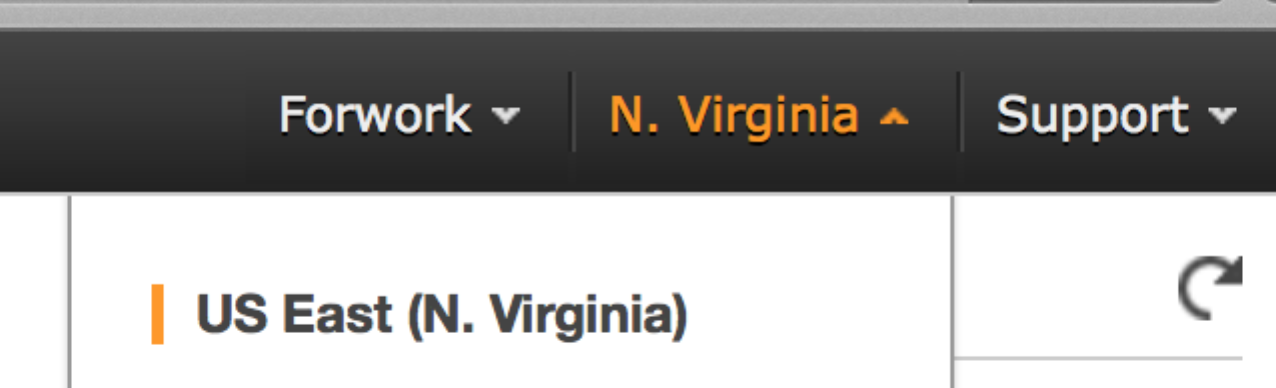
as your region.


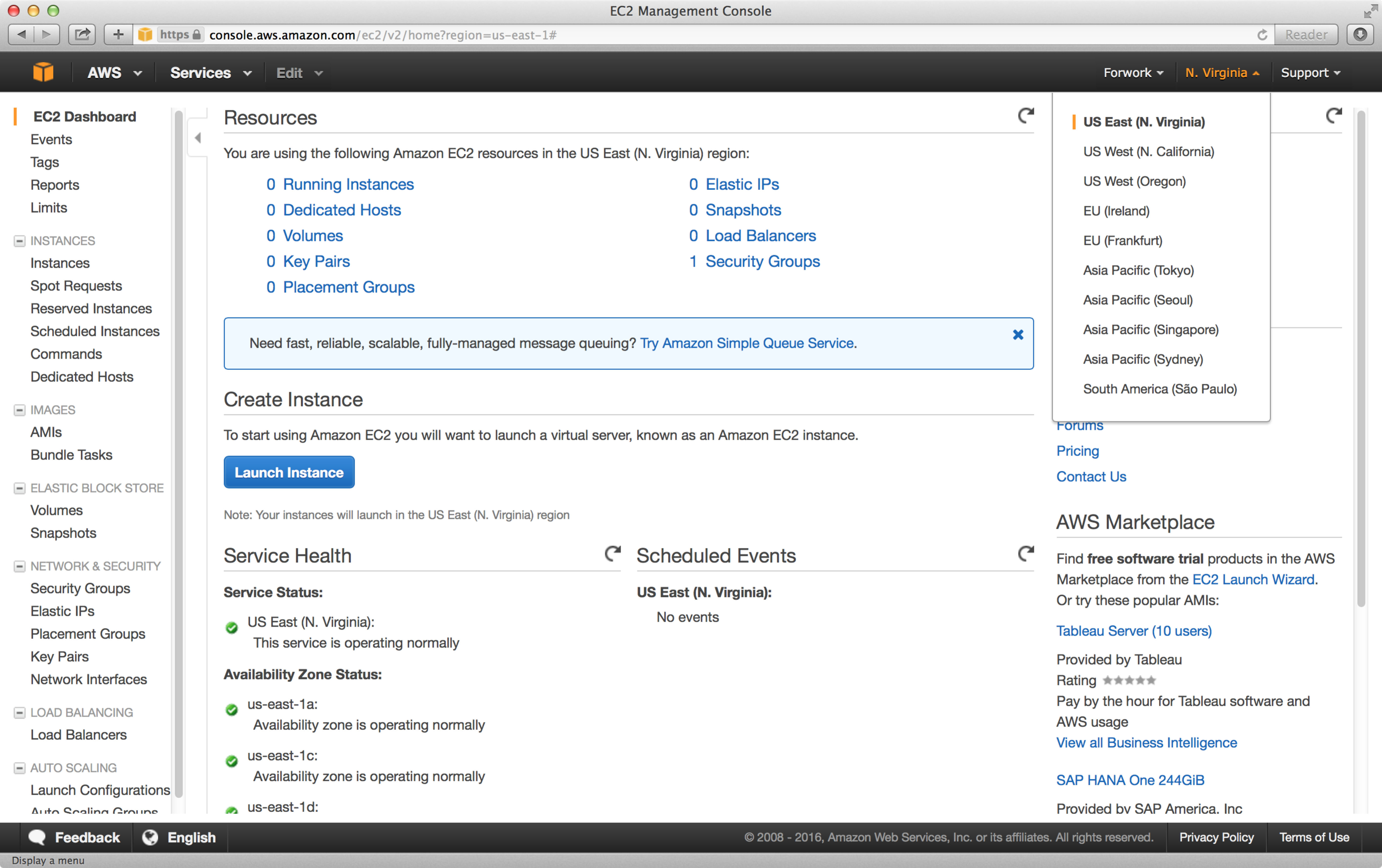


5. Click “AMIs” under “Images”
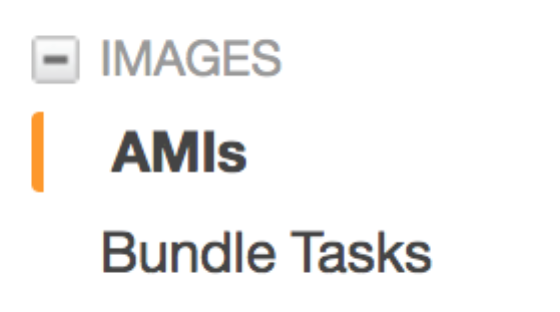
 menu option.


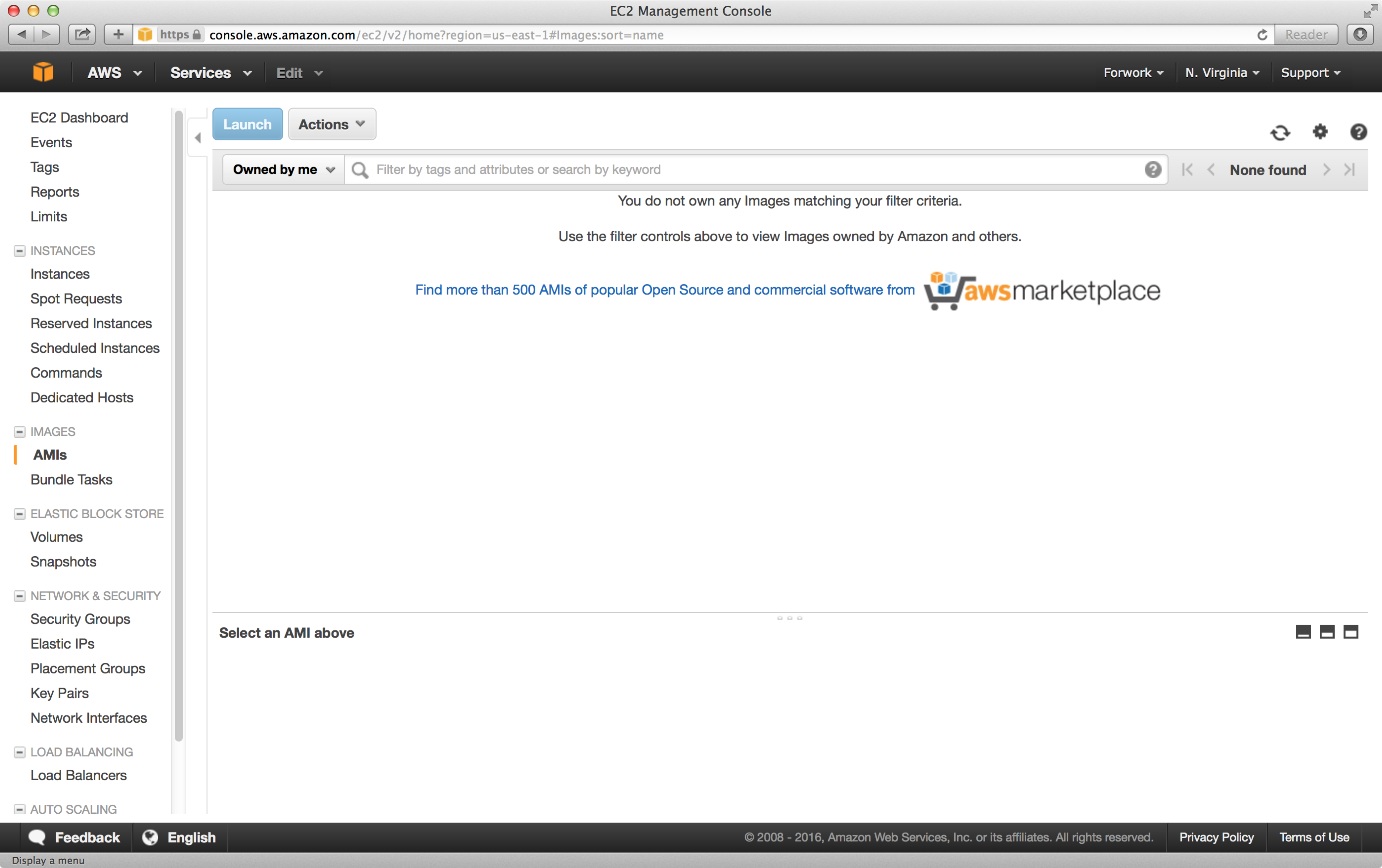


6. Now, click “Owned by me” and choose “Public images.”
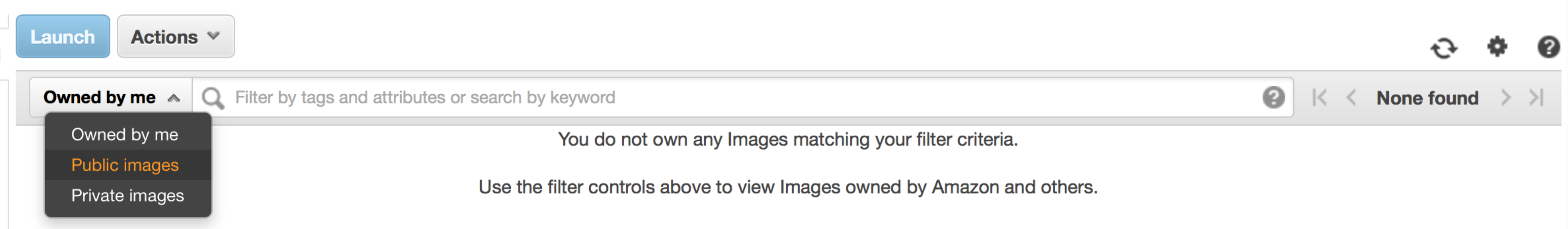


7. With “Public images” option set, type “ami-4510312f” and hit “Enter.”


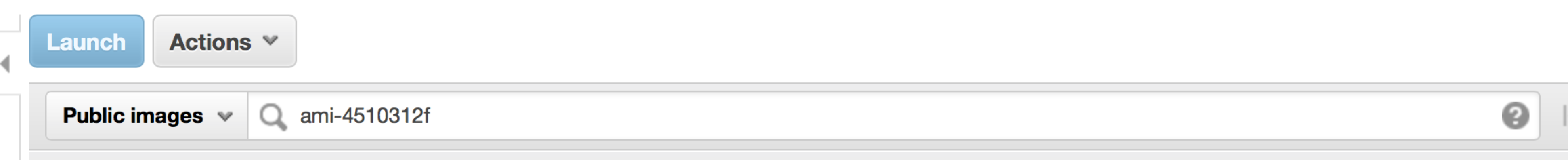


8. You will now see the NCBI-Hackathon-20160122-Network-SNPs AMI. With the Network-SNPs AMI select (by default), hit the “Launch” button to launch a new instance of this machine
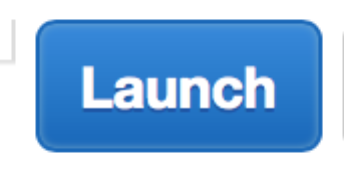
.


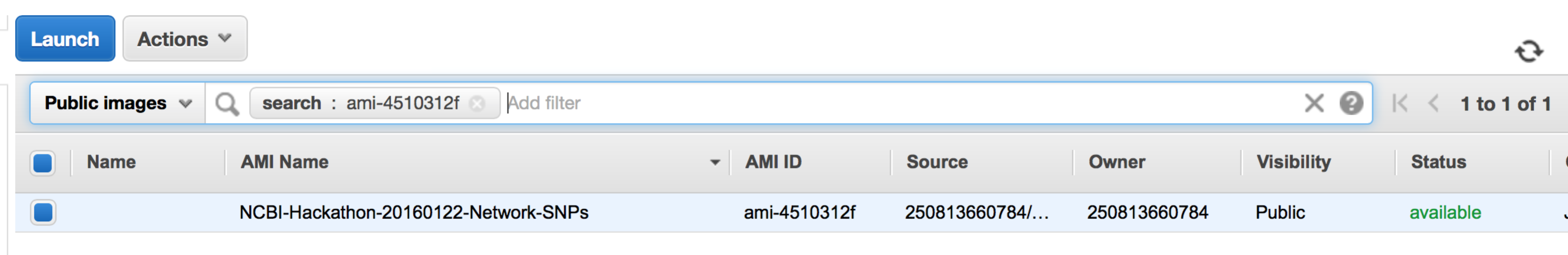


9. For testing purpose, choose the instance type m4.10xlarge. Hit the “Review and Launch” button
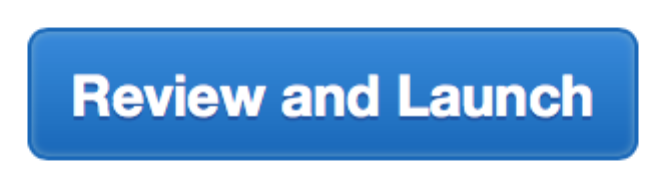
.


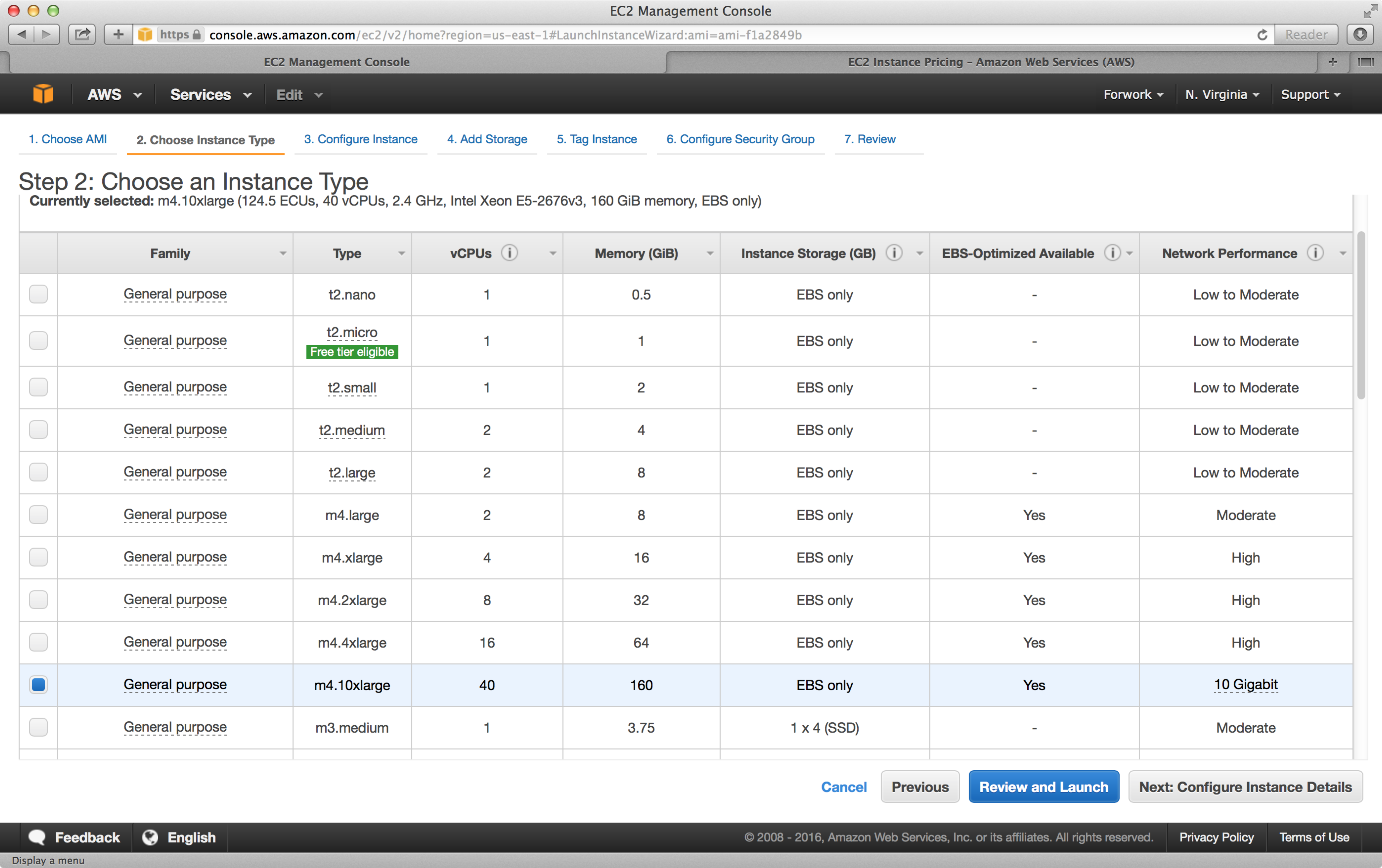


10. You will be presented with warnings about security and free tier usage. You have to fix the security first. Click “Edit Security groups.”
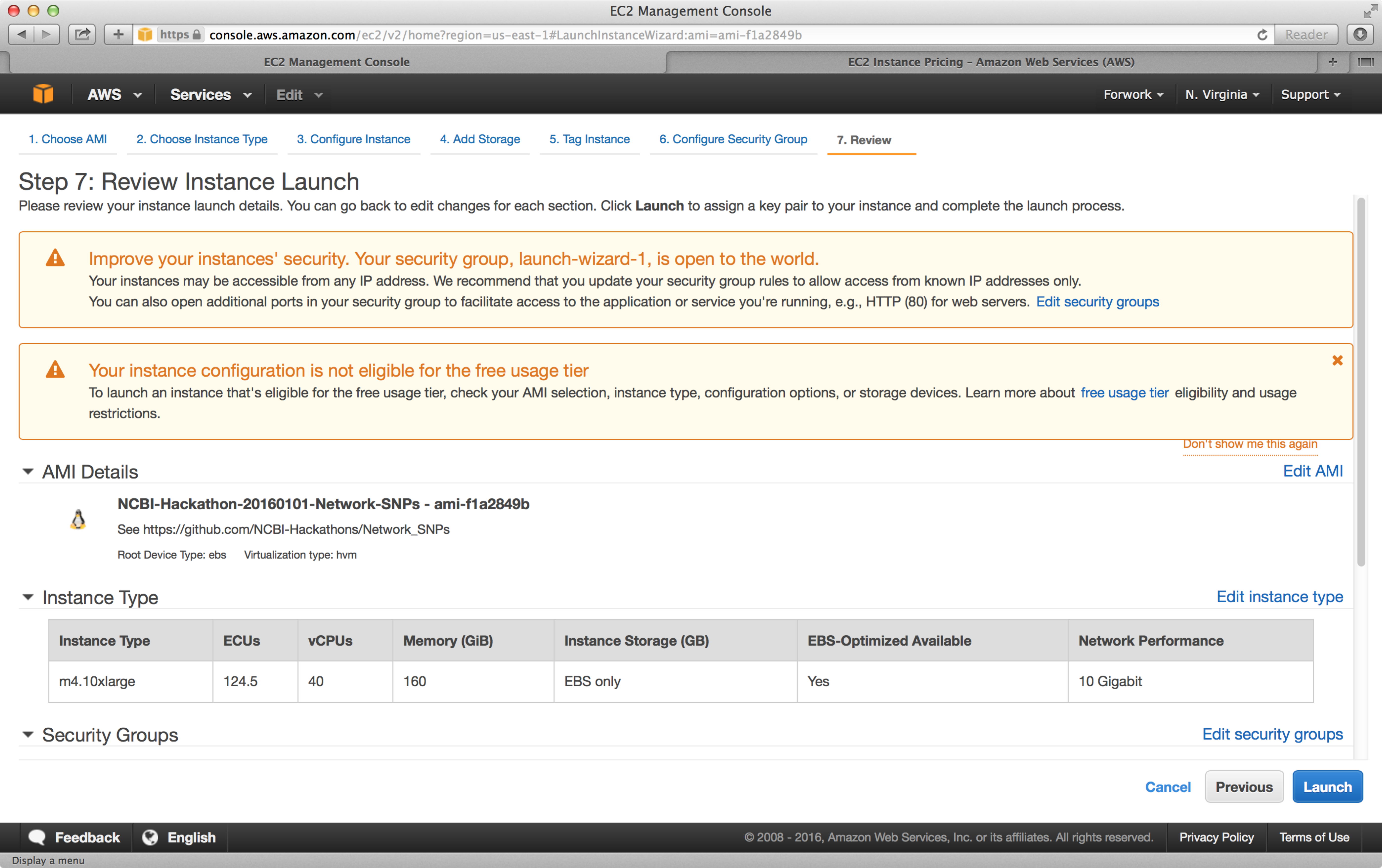


11. For this testing exercise, let’s start by letting only *you* have access the instance, from *your* computer. Set the source to “My IP,” give the group a name (for example, ncbi or anything else you’d like) and hit the “Review and Launch” button
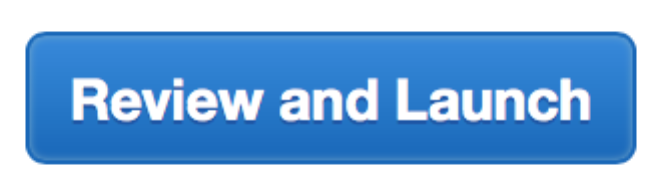

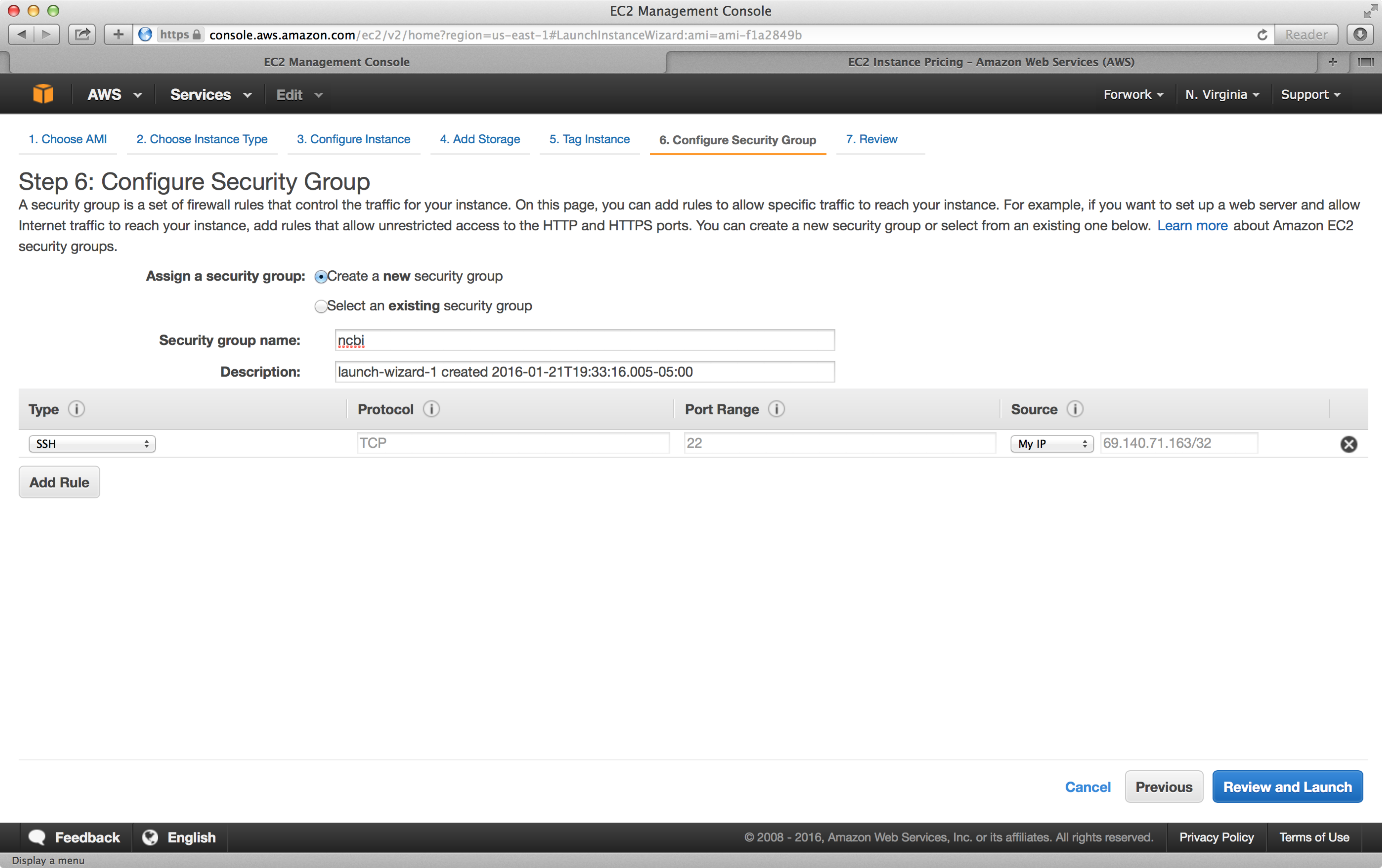


12. In the Step 7 window, hit the “Launch” button
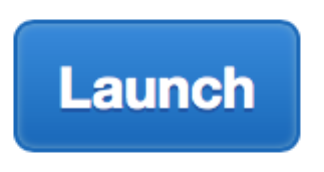
. Take note that you will be charged a fee for the usage of this instance. Though the fee is minimal, please read and understand Amazon’s pricing rules before you do this.
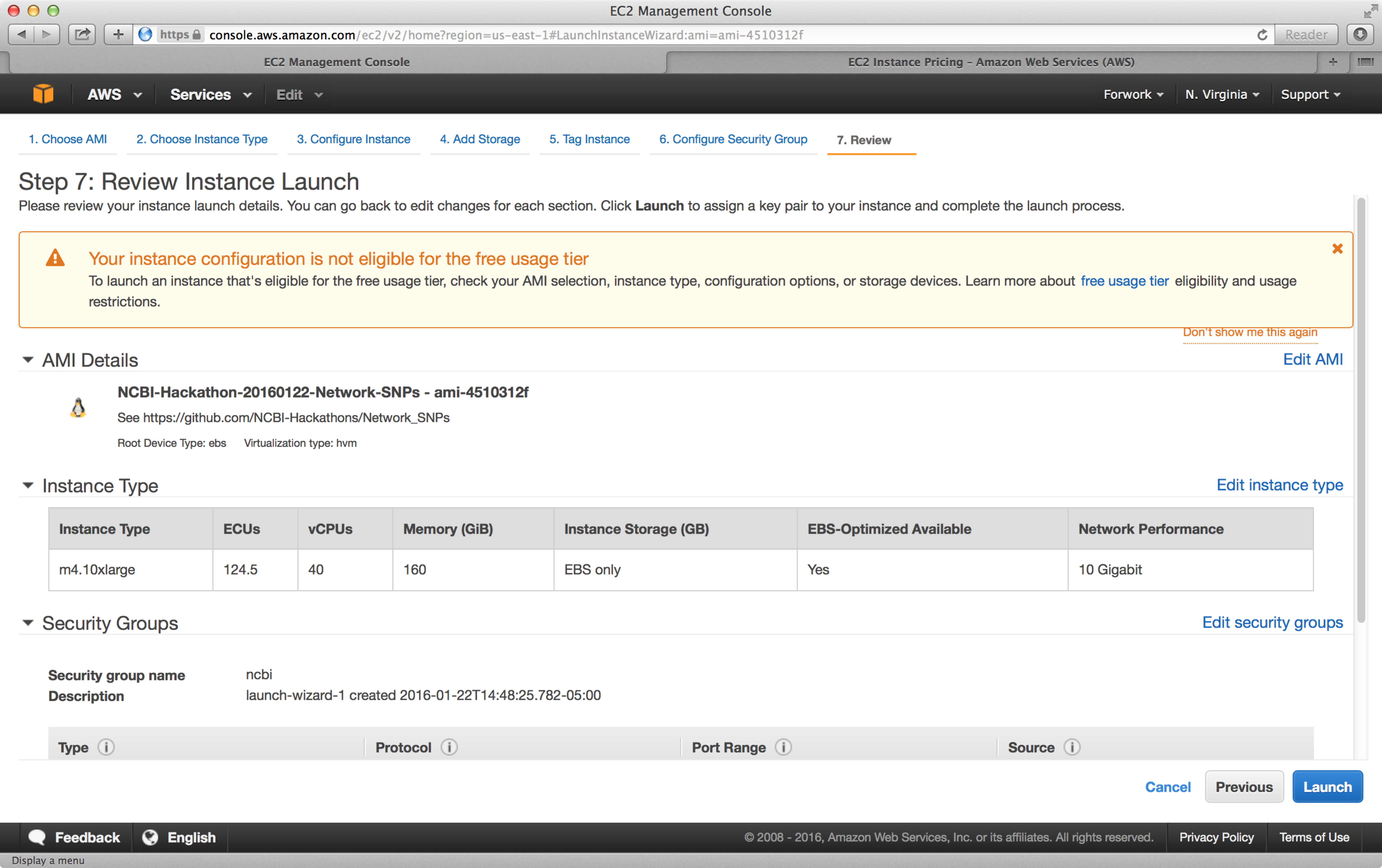


13. Choose “Create a new key pair” in the window, give it a name, and hit the “Download Key Pair” button. Then, hit the “Launch Instances” button.


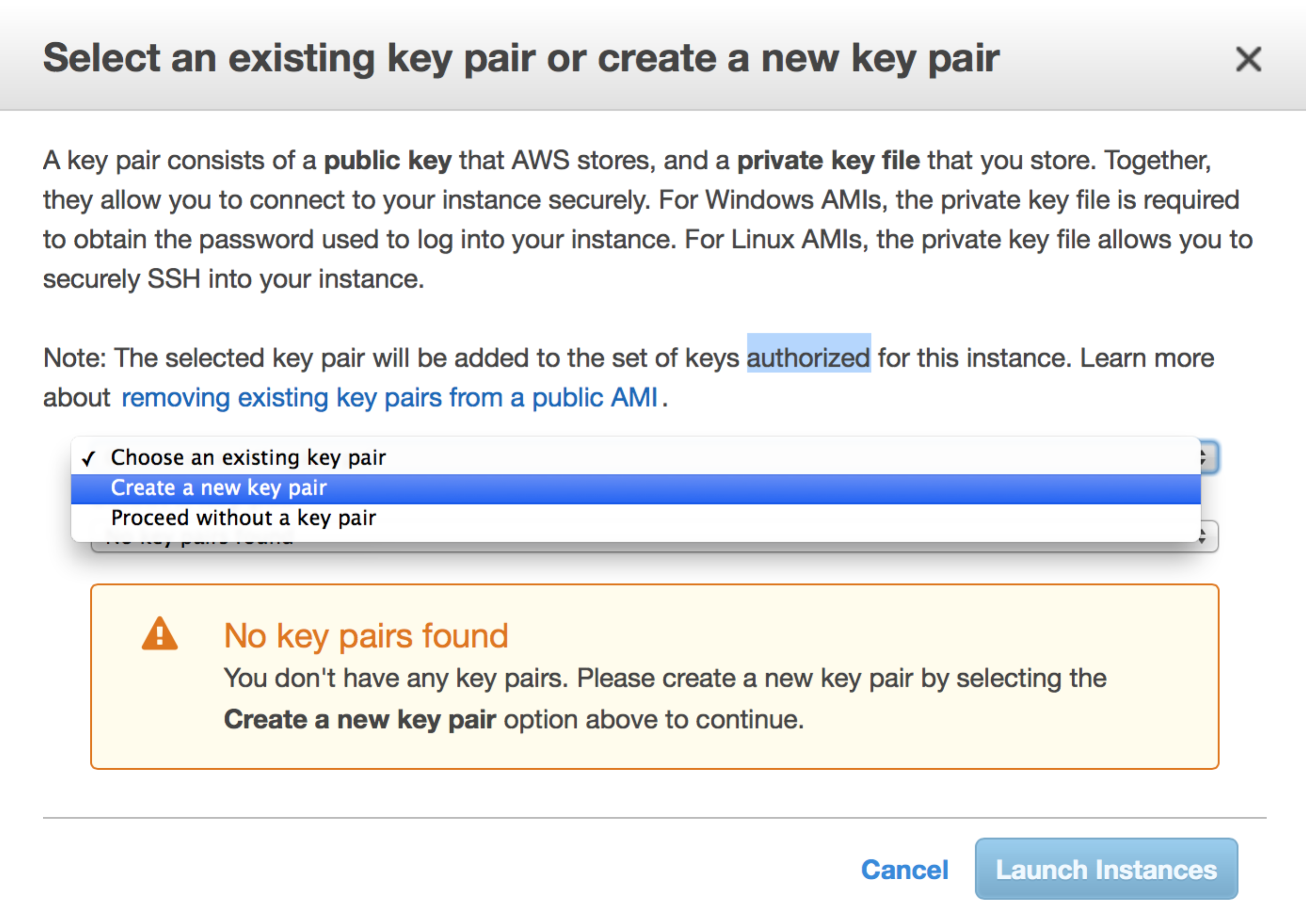

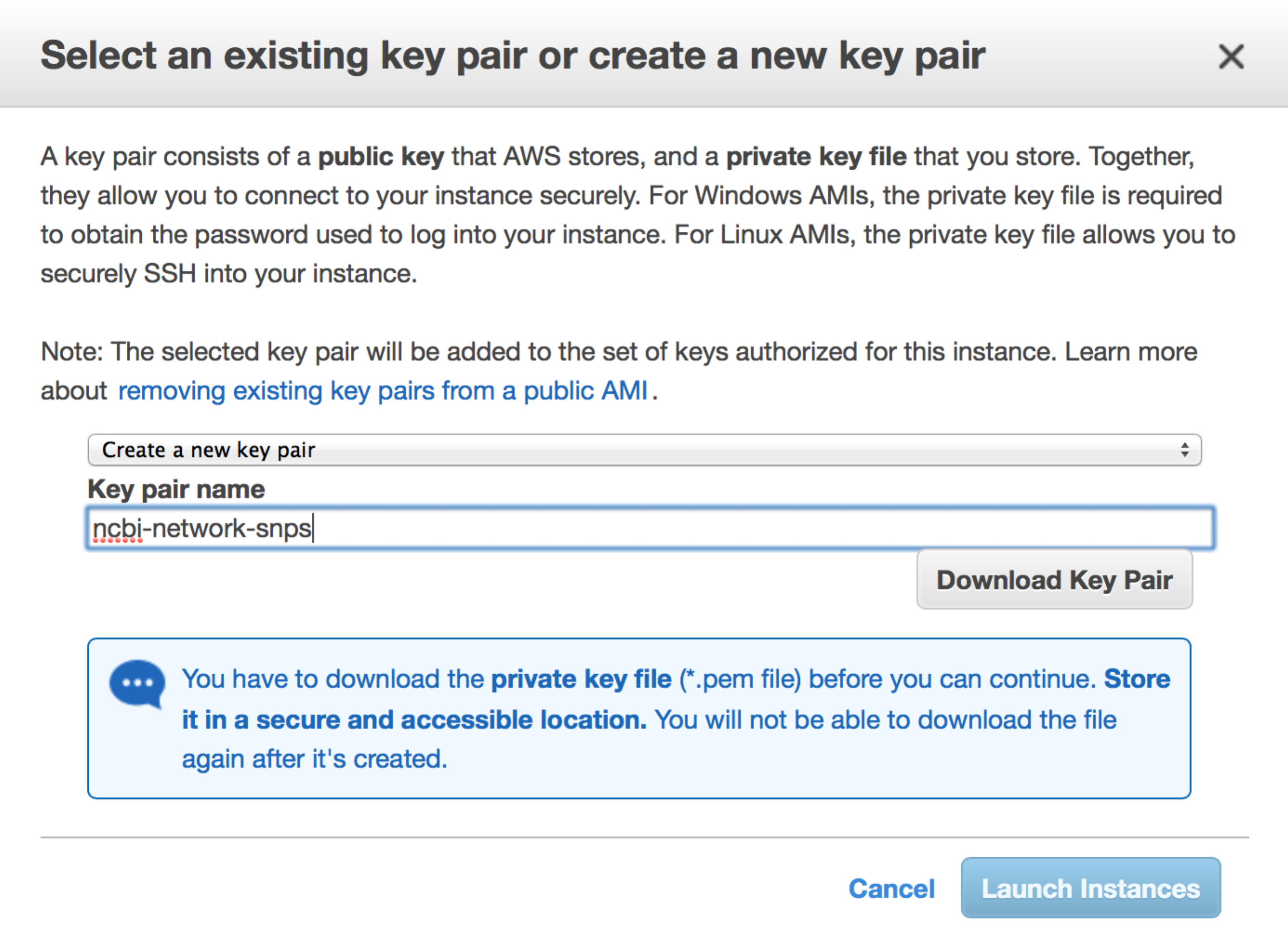


14. If everything has been completed correctly, you will see the following screen, saying that your instance is up and running.


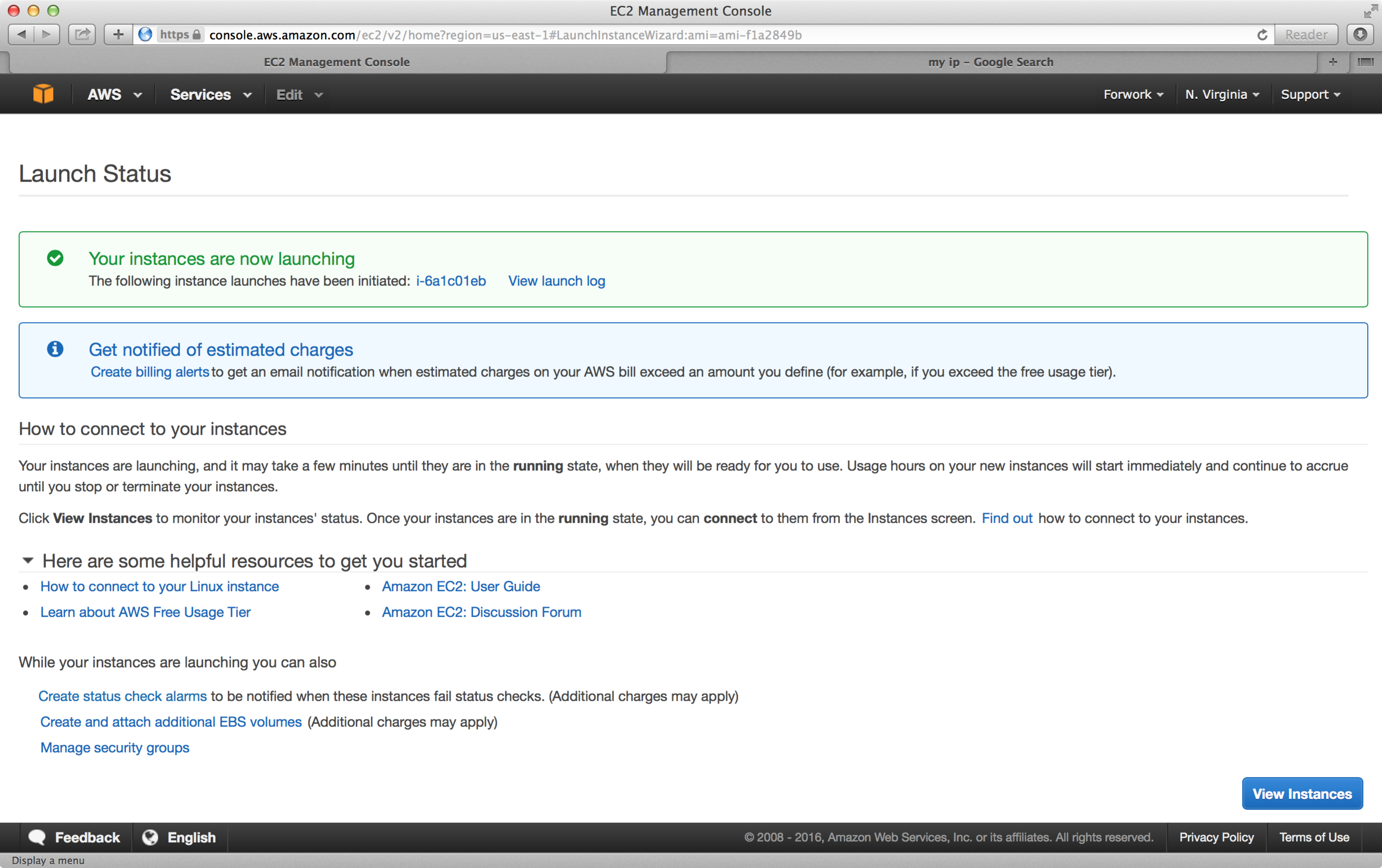


15. Click the “View Instances”
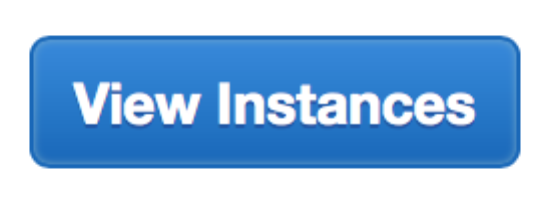
 button in the above screen to go back to the “Instances” menu of the EC2 dashboard, where you will see the following message after a brief delay.


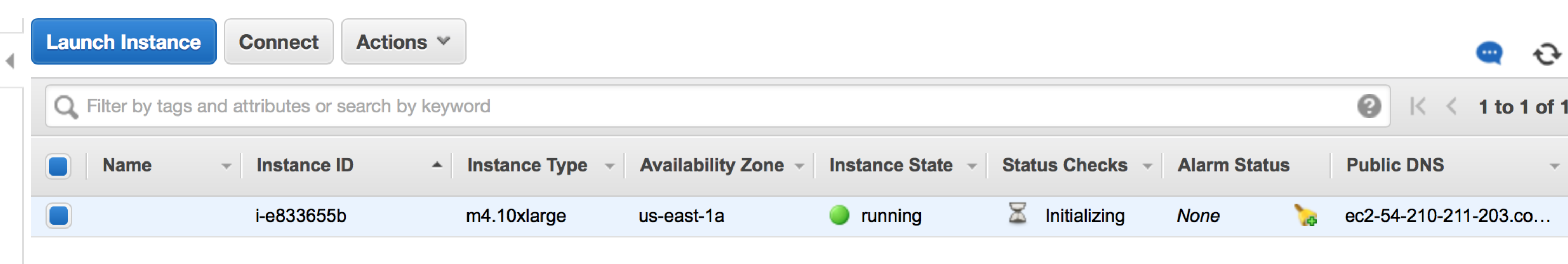


16. Click the “Connect” button in the above screen to get information about how to connect to your instance (this tutorial will also walk you through these steps).


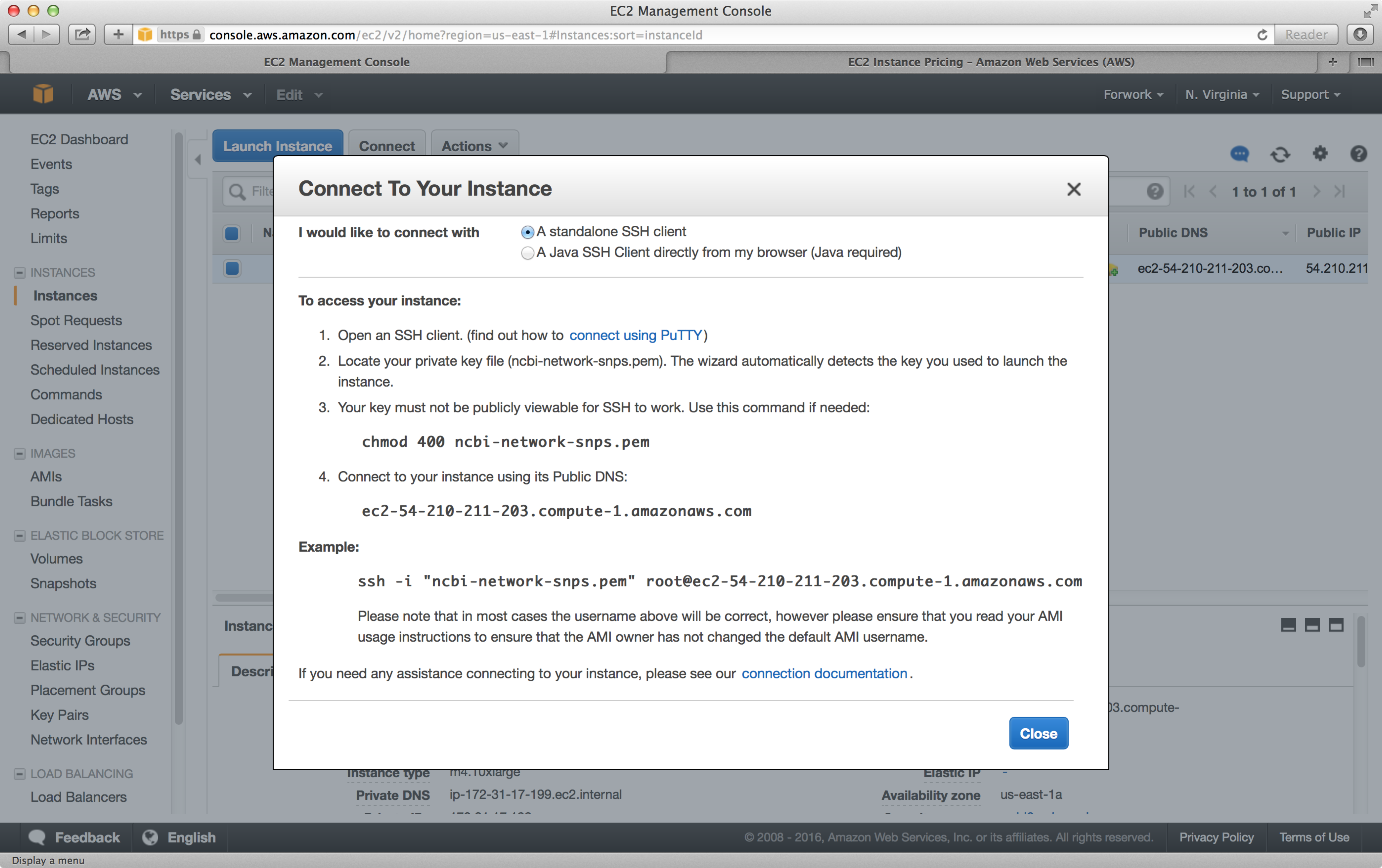


17. In the command line (Mac) go to the directory where you downloaded the private key and chmod 400 that file.


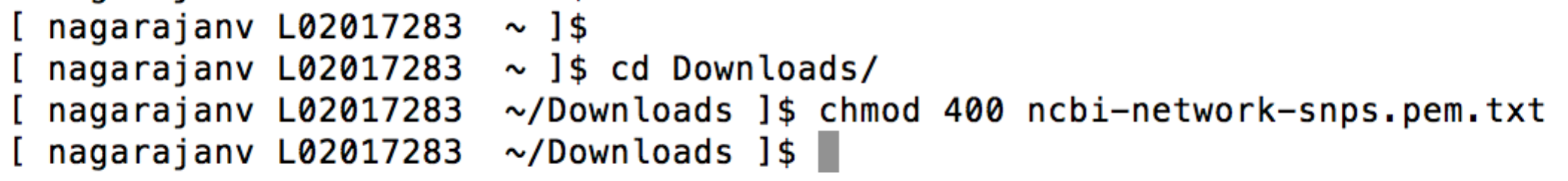


18. Replace the IP address in the below example with the Public IP address for your instance. Now login to the instance using the command “ssh –I “ncbi-network-snps.pem.txt” ubuntu@52.90.111.47.” When prompted, confirm with “yes” to add the key and press enter. Replace the IP address in the above example with the Public IP address for your instance.


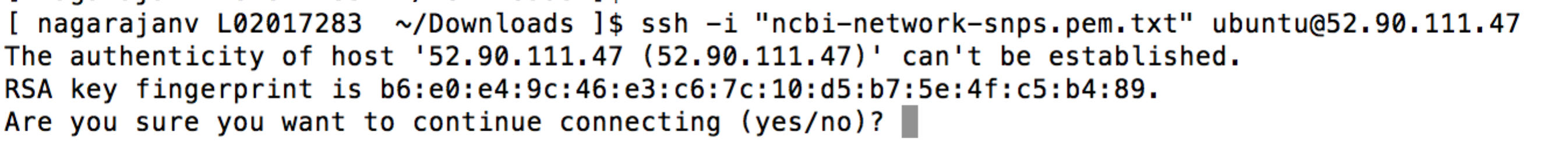


19. A successful login will result in the following screen.


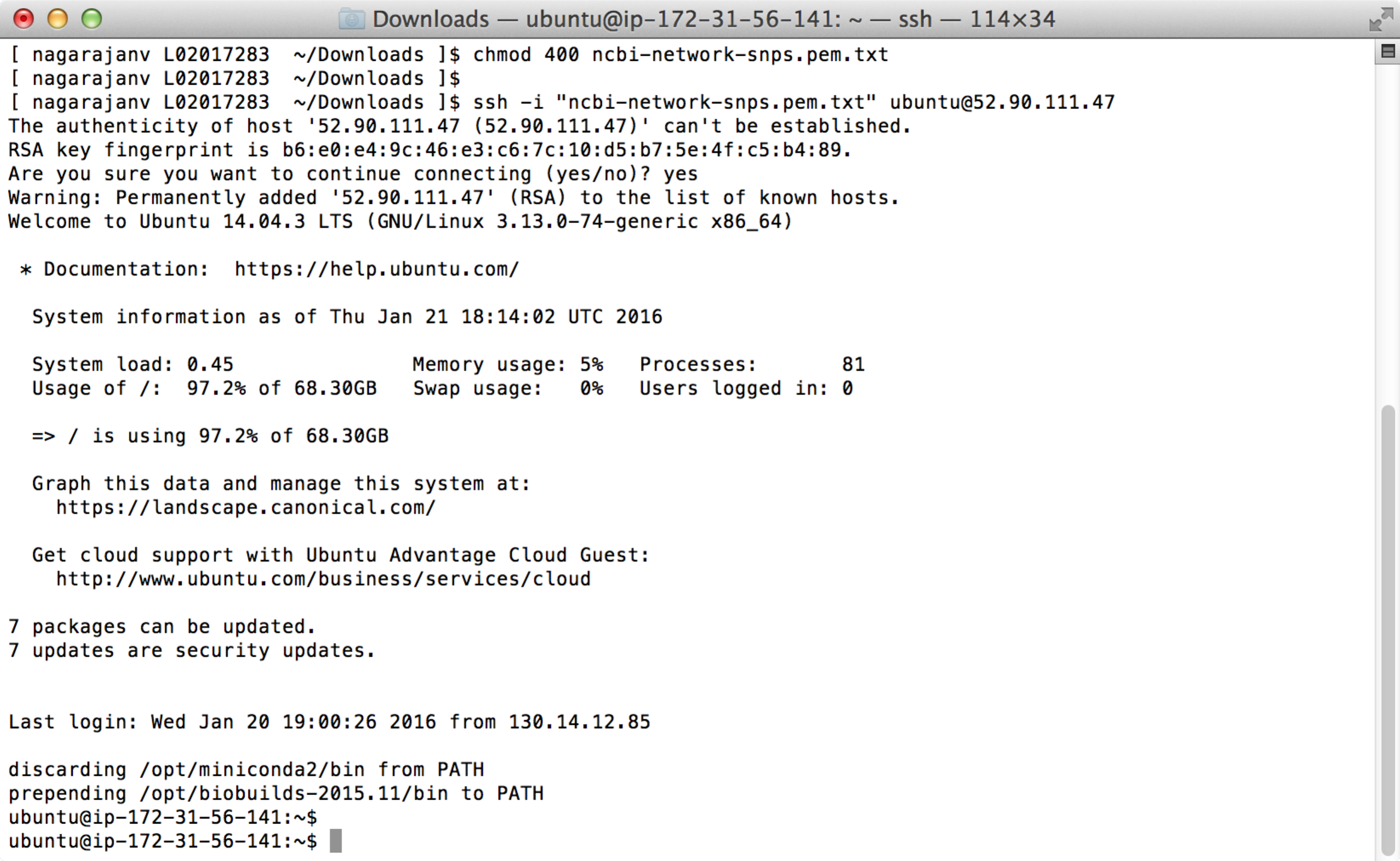


20. Now we are ready to run the pipeline…

Change to “eric/Network_SNPs” directory (command : “cd eric/Network_SNPs”),


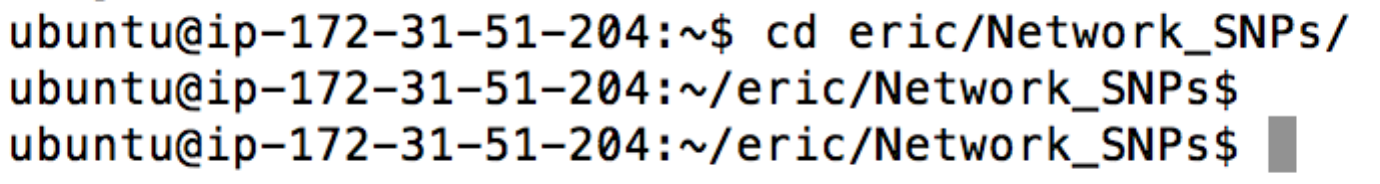


21. MetaNetVar pipeline-related files are in this Network_SNPs directory. This is the directory with contents last pulled from the respective GitHub page. Type “ls” to list files in the directory, and make sure you see the below file.


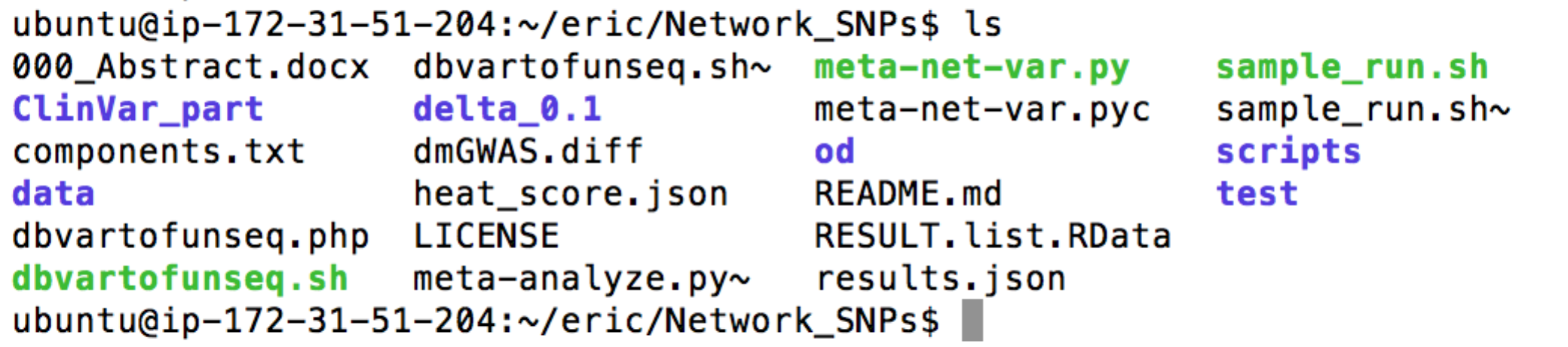


22. “sample_run.sh” is the sample test file for the pipeline. Run this test by typing the command ./sample_run.sh at the command line and hitting Enter. If the test is successful, the pipeline will start running and you will see the screen like below.


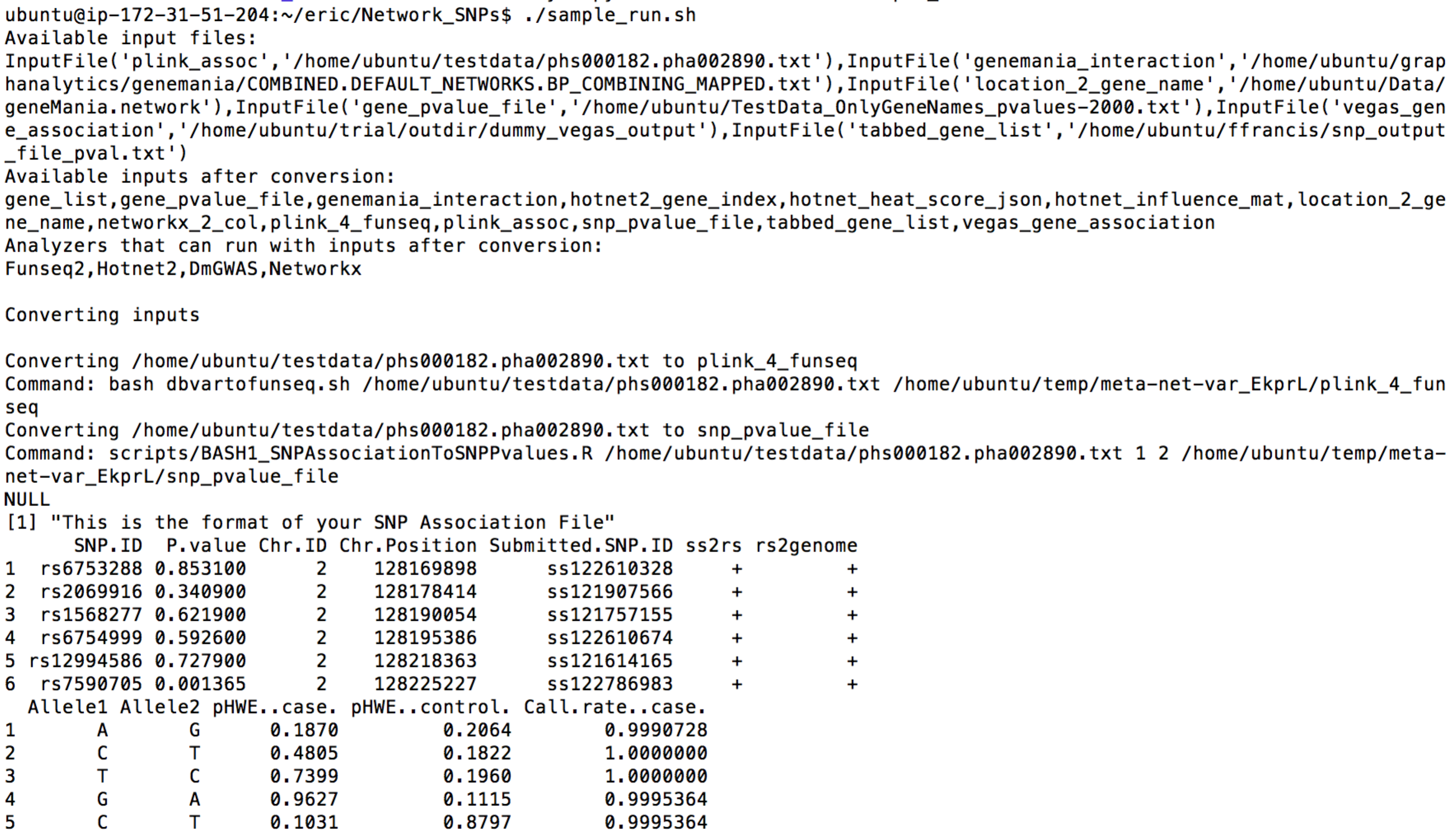


23. It will take about 30 minutes for this sample run to complete. You can check your EC2 dashboard for status. The m4.10xlarge instance costs only a few dollars per hour, so your sample run should cost about $3 one run, as long as you stop the instance after your run. Please check Amazon conditions for any other potential costs (including storage and other features used).

24. Once the run is complete, you can stop the instance (screenshot below), so you don’t get charged for computing. You can always start it again later. There might be charges associated with storing the snapshot of your instance (check Amazon for details). If you don’t want to get charged, you could terminate the instance. Also note, if you store anything in AWS, you will get charged for it– again, check Amazon for details.


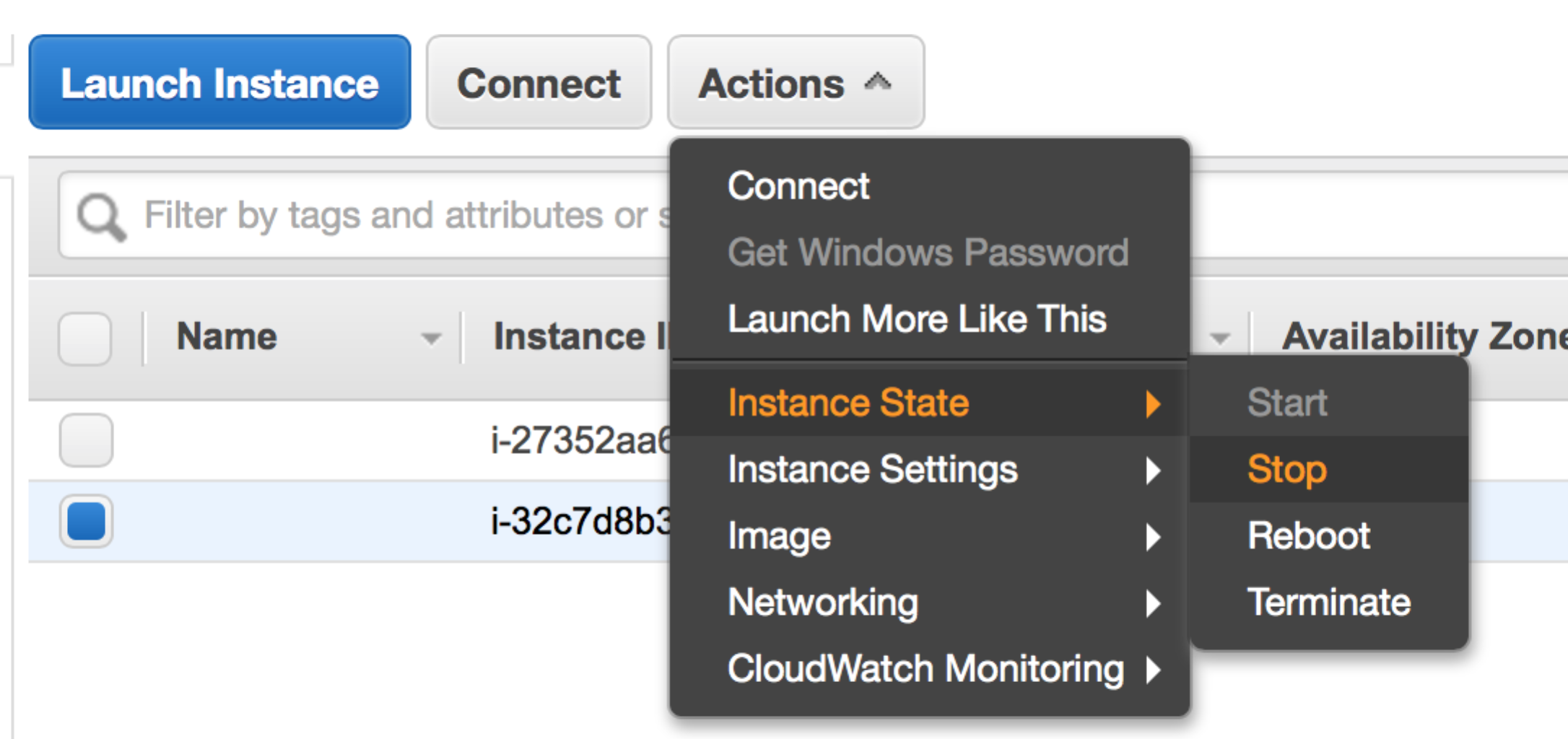


25. If the pipeline runs without any errors, your results will be in the “trial/outdir” folder in your home directory, in four folders, each for one component of the pipeline (screenshot below). Refer to the manuscript or the pipeline’s GitHub page (<https://github.com/NCBI-Hackathons/Network_SNPs>) for more information on the pipeline and individual components.


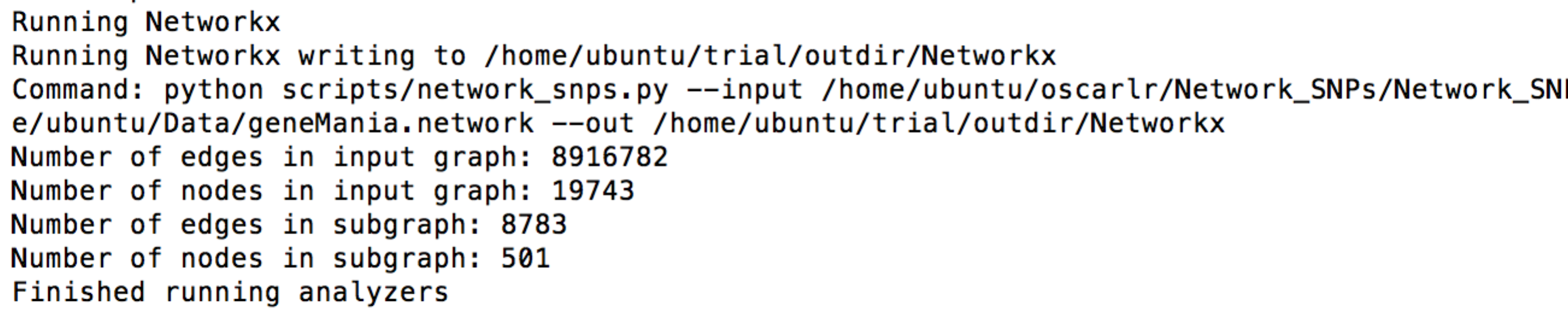


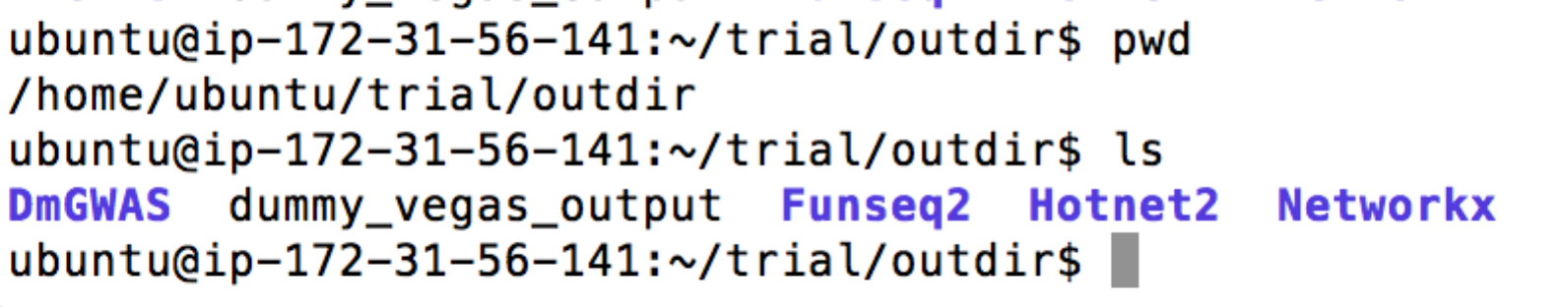

Supplement: Supplementary file 1 [file f1000research-5-8914-s0000.tgz › 13481631-6948-4e98-81f8-08d41f2612e1.docx]
