# Supplementary material for: MetaNetVar: Pipeline for applying network analysis tools for genomic variants analysis
Source: F1000Res. 2016 Apr 13;5:674. [Version 1] doi: 10.12688/f1000research.8288.1 (PMC4857755; doi:10.12688/f1000research.8288.1)
Supplement: Peer review history

# Peer review: Referee response for version 1

**DOI:** 10.5256/f1000research.8914.r13366

**Published:** 2016-04-29

**Authors:** Tomasz Adamusiak

**Affiliations:** 1 Thomson Reuters, Boston, MA, USA

Excellent work given the limited hackathon time frame. I commend the authors for providing an AMI image and source code for the project.

Minor comments:

- *aiding in this problem is network analyses.*
  Should be analysis
- Would change AWS deployment manual format to pdf and provide instructions how to stop the EC2 instance so that the user doesn't not incur unnecessary costs.

I have read this submission. I believe that I have an appropriate level of expertise to confirm that it is of an acceptable scientific standard.

# Peer review: Referee response for version 1

**DOI:** 10.5256/f1000research.8914.r13368

**Published:** 2016-04-26

**Authors:** Sahar Al Seesi

**Affiliations:** 1 Department of Computer Science and Engineering, University of Connecticut, Storrs, CT, USA

The authors describe a pilot version of an integrated pipeline of network analysis tools for genomic variants. It includes four existing tools. The pipeline analyzes the input files and run the tools applicable to the input files. The value of this contribution would greatly increase if the pipeline consolidated the output of the different tools. The authors acknowledge this fact and plan to include that in future versions of the pipeline

The manuscript is well written, and the functionality of tools included is clearly described.

I have read this submission. I believe that I have an appropriate level of expertise to confirm that it is of an acceptable scientific standard.

# Peer review: Referee response for version 1

**DOI:** 10.5256/f1000research.8914.r13367

**Published:** 2016-04-21

**Authors:** John Didion

**Affiliations:** 1 National Human Genome Research Institute, National Institutes of Health, Bethesda, MD, USA

Minor points:

- "...somewhat inaccessible to users with weaker computational backgrounds" - I have a strong computational background, and dealing with poor build processes and user interfaces is frustrating to me also. Maybe rephrase this to say that different tools have different levels of usability, which can be ameliorated by providing a single, well-designed interface to multiple tools.
- In Figure 1, "Network-based variant analysis tools" is represented as a single box, but there are multiple steps encapsulated there. It would be more informative to show the steps involved for each of the four tools and the output of each tool.

I have read this submission. I believe that I have an appropriate level of expertise to confirm that it is of an acceptable scientific standard.
